# Case Report: Gastric Metastasis revealing a Disseminated Skin Melanoma: A Case Report and Literature Review

**DOI:** 10.12688/f1000research.155815.1

**Published:** 2024-09-06

**Authors:** Ramzi Tababi, Amal Khsiba, Moufida Mahmoudi, Asma Ben Mohamed, Manel Yakoubi, Ghada Gharbi, Abir Chaabane, Emna Chelbi, Mouna Medhioub, Mohamed Lamine Hamzaoui

**Affiliations:** 1 Gastroenterology Department, Mohamed Taher Maamouri University Hospital Nabeul, Mrezga, Nabeul, Tunisia; 2 Pathology Department, Mohamed Taher Maamouri University Hospital Nabeul, Mrezga, Nabeul, Tunisia

**Keywords:** Melanoma, Gastric metastasis, Multi-organ metastases, Digestive endoscopy, Histopathology

## Abstract

**Background:**

Melanoma, an aggressive malignant skin cancer, has the ability to spread both locoregionally and to distant sites. The risk of metastasis is correlated to invasion depth and the presence of ulceration. Although gastrointestinal (GI) metastases are uncommon, gastric involvement is particularly rare.

**Case presentation:**

We report a case of a 62-year-old male who presented with abdominal pain, dyspepsia, anorexia, and weight loss. On physical examination abdominal masses and hepatomegaly were detected. Radiological imaging showed widespread masses in the abdominal and thoracic regions. Upper GI endoscopy identified an umbilicated protruded lesion with central dark pigmentation at the antro-fundic junction. Histopathological examination and immunohistochemical staining were consistent with melanoma. A subsequent rigorous skin examination uncovered a primary malignant skin melanoma. Due to worsening general condition, the patient received palliative hospice care.

**Conclusion:**

This report highlights the critical need for vigilant skin examination when encountering gastric lesions with dark pigmentation, which led to the identification of initially undetected cutaneous melanoma.

## Introduction

The incidence of melanoma is on the rise, particularly in developed countries with lighter skin populations, representing 1.7% of global cancer cases. ^1^ It also constitutes 10% of all skin cancers and remains the primary cause of death among these malignancies. ^2^ In fact, melanoma embodies a heterogeneous tumour group of distinct precursor cells, biological signature and presentations. ^1^^,^^2^ Nodular melanoma is notably a malignant subtype, known for its aggressive behaviour and accounting for 16% to 25.6% of invasive cutaneous melanoma. ^2^

The most frequent metastatic sites include the skin, lungs, liver, central nervous system and bones. Less frequently, metastases occur in the kidneys, adrenal glands, and gastrointestinal (GI) tract, with gastric involvement being particularly rare. ^3^

In this report, we present a rare case of gastric metastasis from melanoma that led to the discovery of the initially unnoticed primary skin lesion. We describe the endoscopic and histopathological findings, as well as the nonspecific clinical symptoms that prompted the diagnostic investigation.

## Case report

A 62-year-old Caucasian male patient, with a medical history of hypertension and coronary artery disease, presented with diffuse abdominal pain, along with dyspepsia, anorexia and weight loss for the past 6 months. Physical examination revealed abdominal tenderness, as well as multiple fixed and hard nodules of the thoracic and abdominal walls, as well as in the Douglas pouch on rectal examination, suggestive of carcinomatosis. An enlarged, firm, and tender liver was also noted. No jaundice or palpable lymph nodes were observed.

Laboratory findings were within the normal range, notably for complete blood count and liver function tests.

**Figure 1.**
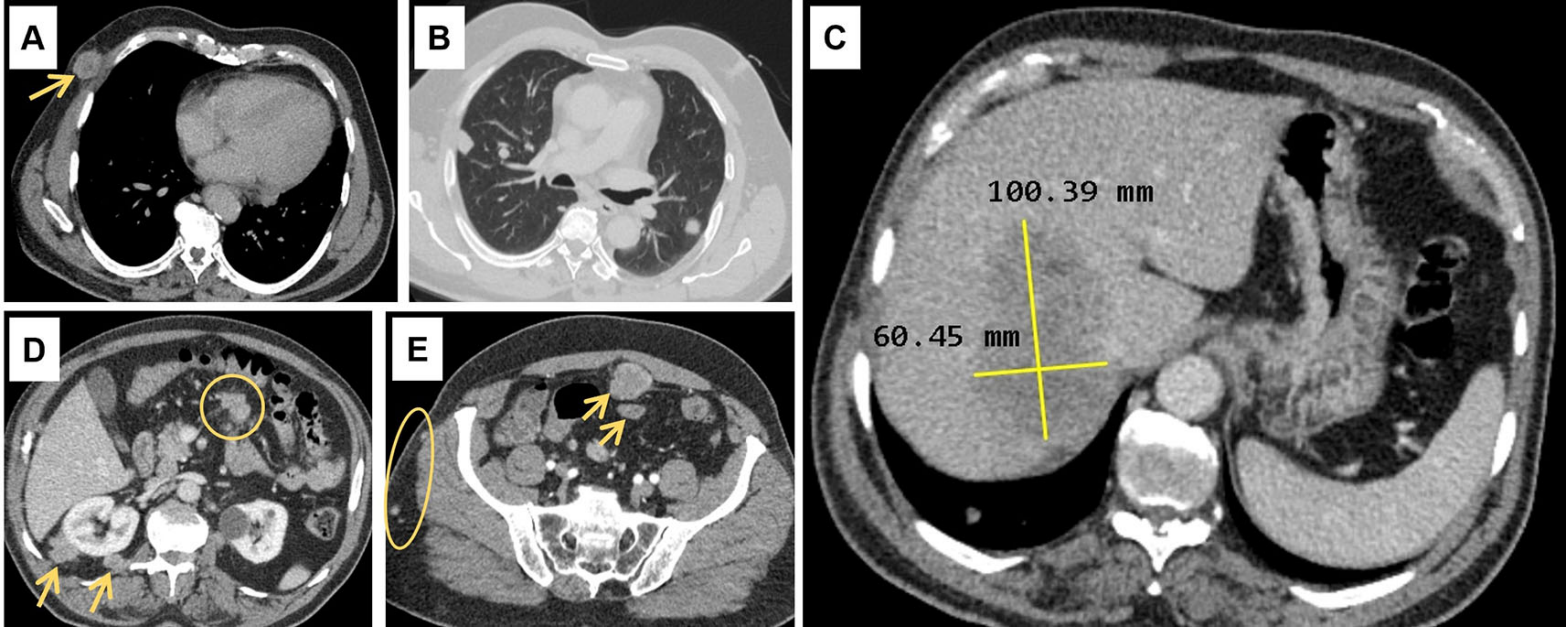
Computed tomography scan findings. (A): Subcutaneous soft tissue nodule of the anterior right thoracic wall (arrow). (B): Bilateral pulmonary nodules. (C): Hypodense hepatic mass in the right lobe on the portal phase, measuring 100 × 60 millimeters. (D): Intraperitoneal adenomegalies (circle) and heterogeneous nodules in the right perirenal retroperitoneal region. (E): Heterogeneous peritoneal nodules (arrows) and small subcutaneous soft tissue nodules in the right lateral abdominal wall (circle).

Abdominal ultrasound (US) showed a non-dysmorphic liver with a heterogenous lobulated hypoechoic mass, vascularised on Doppler, measuring 83 × 60 mm, associated with omental adipose tissue nodule of 37 mm, sharing the same characteristics. On the CT scan ( Figure 1), the hepatic mass was isodense, poorly defined, spanning segments V, VII and VIII, showing weak enhancement with contrast, measuring 100 × 60 mm. There were also multiple scattered tissue masses enhanced with contrast, located in the peritoneum, in the retroperitoneum, and sub-peritoneal, as well as in the left adrenal gland. The pancreas was normal. Plus, multiple solid pulmonary nodules were observed, involving all segments, alongside mediastinal and abdominal lymphadenopathies.

At this point, widespread metastases were the leading considered diagnosis based on the clinical context and radiological presentation. Alpha-fetoprotein and prostate specific antigen levels were normal.

**Figure 2.**
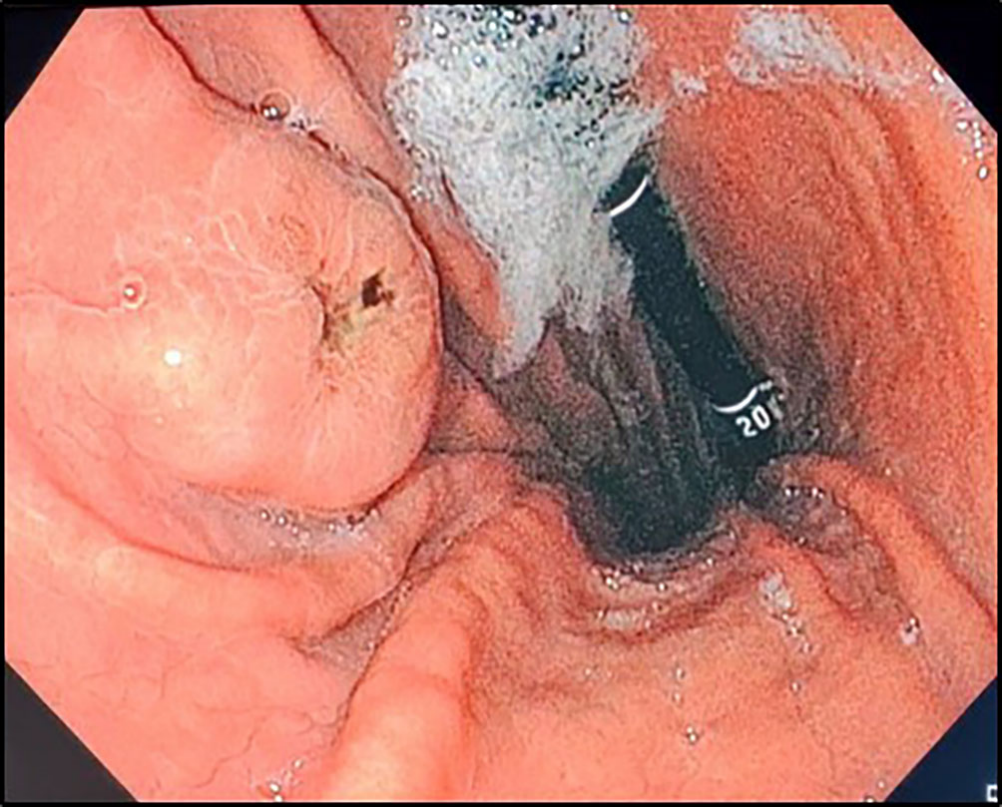
Upper gastrointestinal endoscopy findings. Retroflexion shows an elevated lesion at the antrum-fundus junction with a depressed centre, resembling a volcano appearance, with brown pigments.

**Figure 3.**
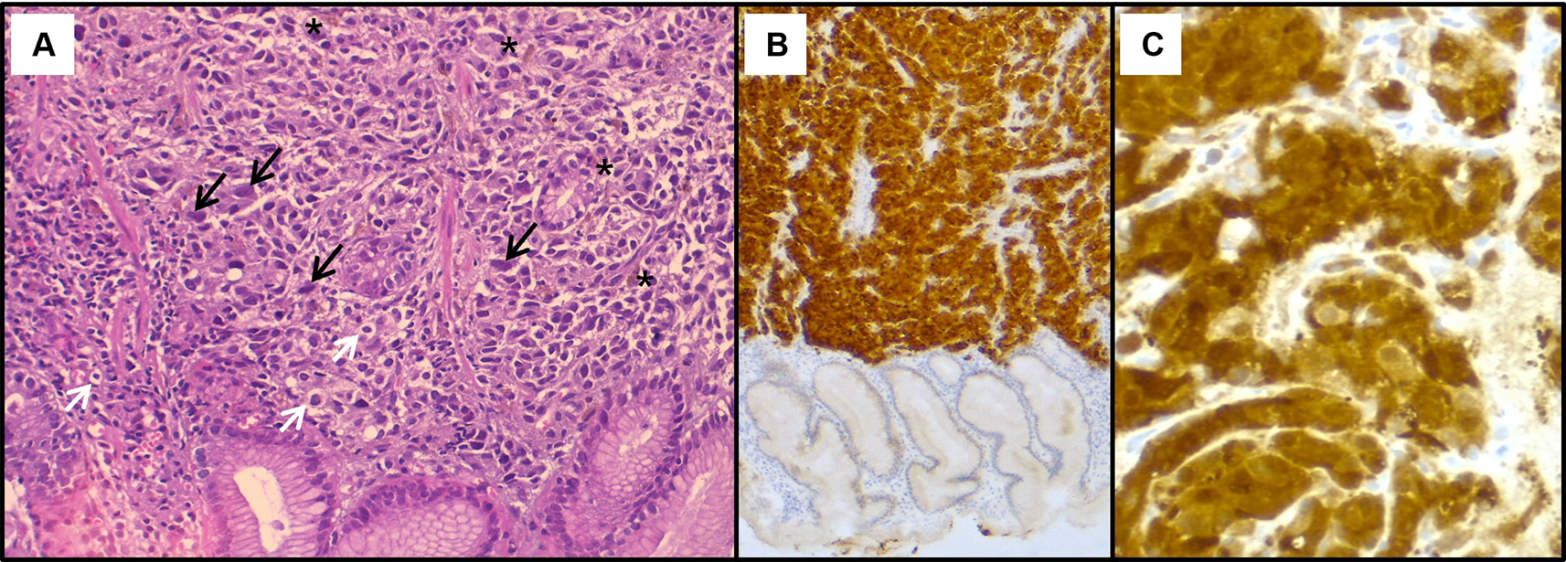
Histopathological findings of the gastric lesion biopsy. (A): Hematoxylin & eosin staining on x200 magnification shows malignant proliferation infiltrating the gastric mucosa. Tumour cells are pleomorphic and disaggregated, exhibiting marked nuclear atypia with hyperchromatic and sometimes monstruous nuclei (black arrows). Some larger cells with clear cytoplasm were present (white arrows). Deposits of brown pigments are also seen (*). Immunohistochemistry demonstrated positive staining with Melan-A (B) and PS 100 (C).

In the investigation for the primary cancer, and given the recent dyspepsia and epigastric pain, an upper gastrointestinal endoscopy was performed revealing a polypoid lesion at the antro-fundic junction measuring 20 mm in diameter, with a depressed and ulcerated centre, containing dark pigmentation ( Figure 2). The histological examination showed a tumour proliferation extensively invading the antro-fundic mucosa, formed by clusters of flattened and disintegrated cells. The tumour cells were frequently small, with hyperchromatic nuclei. In certain areas, larger cells with ballooned, clarified, or vacuolated cytoplasm were seen. Some cells displayed nuclear monstrosity, and deposits of brownish pigments were also noted. Immunohistochemistry analysis revealed positive staining for melanocytic markers Melan-A, S100 protein and HMB45 ( Figure 3). Other markers were negative, namely pancytokeratin, TTF 1, CD 45, chromogranin, and synaptophysin. These findings were consistent with a gastric localisation of a poorly differentiated tumour with an immunohistochemical profile compatible with a melanoma.

**Figure 4.**
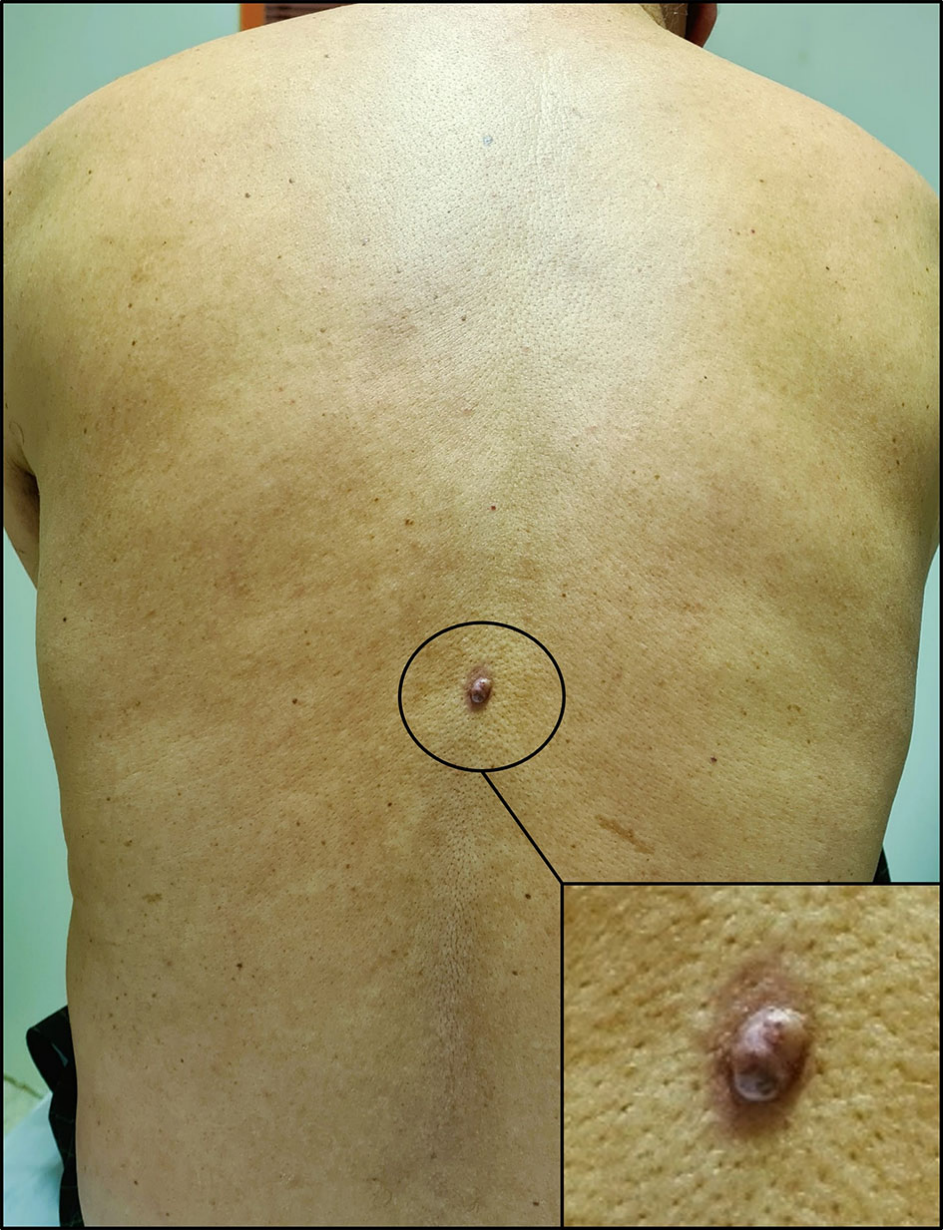
Cutaneous lesion in the back. It appears as a circumscribed dark pink nodule measuring 8 millimeters in diameter with areas of brown discoloration. The lesion also has a slightly irregular surface and a surrounding area of erythema.

With these results in mind, a thorough skin examination was thus undertaken unveiling a cutaneous lesion in the back, manifesting as a circumscribed pink nodule of 8 mm with brown discoloration ( Figure 4).

**Figure 5.**
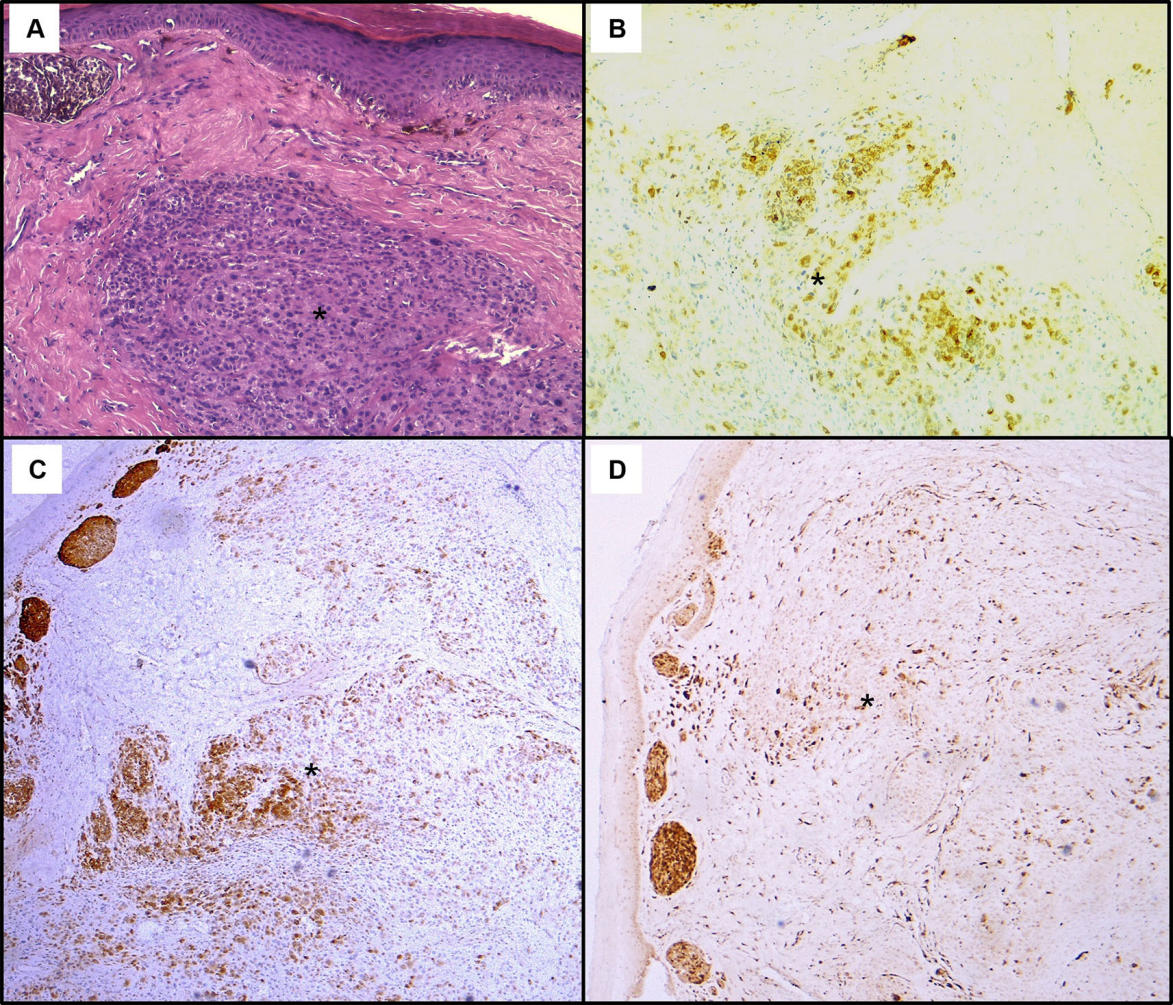
Histopathological findings of the skin lesion biopsy. (A): Hematoxylin & eosin staining on x100 magnification shows malignant cellular proliferation (*) in the dermis. Tumour cells have various size and shape with marked cytological atypia. On immunohistochemistry, they exhibit positive staining with HMB45 (B), Melan-A (C), and p16 (D).

Histological assessment of the skin lesion biopsy showed the presence of a cellular proliferation in the dermis composed of vaguely naevoid cells and larger cells with abundant eosinophilic cytoplasm, atypical pleomorphic nucleolated nuclei and no junctional activity. On immunohistochemistry, tumour cells exhibited positive staining with Melan-A, HMB45, and p16 ( Figure 5). These findings were suggestive of cutaneous nodular melanoma.

Ultimately, the diagnosis of malignant cutaneous melanoma with diffuse multi-organ metastases, particularly involving the stomach, was confirmed. Subsequently, the patient’s general condition deteriorated, precluding the initiation of planned palliative chemotherapy. Supportive care was provided, including pain management, with a documented follow-up of 6 months.

## Discussion

The stomach is an unusual metastatic site, though it can be a localisation of secondary lesions for certain cancers in 2.6% of cases, particularly breast cancer as well as lung cancer and melanoma. ^4^^,^^5^ In fact, melanoma is one of the most frequent malignancies that metastasise in the GI tract. It can spread beyond the primary site to almost all organs. It is hypothesised that tumour cells disseminate through lymphatic nodes to systemic blood circulation via the thoracic duct. ^3^ It has also been noted that cutaneous melanoma preferentially metastasises to upper GI tract, while uveal melanomas are more prone to spread to the liver. ^4^ According to an autopsy study involving 216 patients with advanced malignant melanoma, the GI system was the second most prevalent site of metastases, following the lymph nodes (73.6%) and the lungs (71.3%). ^6^ In this series multiple-organ metastases were most commonly involved (95%). Consistent with these findings, our patient presented disseminated disease affecting the lungs, the liver, the stomach, the subcutaneous tissue, the adrenal gland, the peritoneum, and lymph nodes. Secondary gastric melanoma was observed in 49 cases (22.7%) in that study. The other GI metastases comprised the liver (58.3%), the peritoneum (42.6%), the pancreas (37.5%), the small bowel (35.6%), the spleen (30.6%), the colon (28.2%), the oral cavity and the oesophagus (9.3%), and the biliary tract (8.8%). A previous autopsy series of 100 patients with cutaneous melanoma reported comparable prevalence, with gastric involvement in 26%. ^7^ Although, GI metastases might not seem infrequent in autopsy, they are very likely to be subclinical, given that their incidence in clinical series are less high, namely intestinal metastases for instance which are found in only 1% to 7% of patients. ^3^

Melanoma gastric metastases may either be present at the primary diagnosis or occur even decades later indicating recurrence. ^8^ In our study, the gastric lesion confirmed the melanoma diagnosis and motivated a further physical examination, uncovering a skin lesion that was initially missed during the first assessment. In fact, endoscopy was driven by the presence of upper abdominal discomfort. Nevertheless, metastases to the stomach are rarely detected pre-mortem owing to nonspecific symptoms that could be taken on the account of the general disease burden. As an illustration, the gastric localisation in our case could have been missed if epigastric pain and dyspepsia were attributed by default to a potential extrinsic compression by the liver mass or the peritoneal nodules, eventually sparing the patient upper GI endoscopy. This observation underscores the importance of GI endoscopy for patients with digestive symptoms in the context of malignancy. That can also provide a diagnostic confirmation and spare the patient from more invasive procedures, such as deep organ biopsies.

A recent systematic review by Reggiani et al. ^9^ included 113 patients with gastric metastases of melanoma, predominantly of primary skin site (62%). They were mostly male patients (64%) with a median age of 63 years old. They were asymptomatic in only 10% of cases, however the main symptoms included bleeding (34,5%), abdominal pain (34,5%), anorexia and weight loss (23%), as well as nausea and vomiting (17.7%). Dyspepsia was noted in only 5.3%. Gastric metastases were single in 42.5%, located mainly in the gastric body in 60.2% of cases. Yet, the diverse descriptions of lesions across the included studies resulted in significant variability, preventing the extraction of consensus uniform endoscopic characteristics in this review.

However, an endoscopic classification was proposed by Nelson et al. ^10^ in 1978 which identified three types of GI tract melanoma metastases: the first type comprises melanotic nodules of different sizes generally emerging on the top of normal folds, often with ulcerated tips. The second type consists of an elevated submucosal lesion with ulcerated centre with or without visible melanin, consistent with the lesion observed in our study. This type usually displays the “bull’s eye” aspect on radiological examination. The third type covers mass lesions with different extent of necrosis and melanosis. Plus, the morphology of raised lesions with ulcerated centre and no edge infiltration is often termed as volcano-like or donut-shaped, ^9^ also resembling the lesion’s appearance in our case. Accordingly, such protruding lesions, especially with brown deposits, should suggest the possibility of gastric melanoma. Besides, other endoscopic descriptions have been deployed such as polypoid mass, small nodules, ulcers, and small black spots. ^5^^,^^8^^,^^11^

GI melanoma metastases can also mimic the appearance of primary gastric cancer or other gastric metastases. ^5^^,^^9^^,^^11^ Therefore, histopathological examination along with immunohistochemical staining are key for final diagnosis. These metastases usually exhibit evident melanocytic characteristics, including cytological atypia, pleomorphism, sheet-like growth pattern, and increased proliferative activity, alongside positive staining with melanocytic markers, ^12^ as found in both gastric and skin lesions in the current case. Nonetheless, typical immunophenotypic features can be lost in metastatic or primary lesions during tumour progression, known as phenotypic switching or cellular plasticity, as it has been reported cases of dedifferentiated and undifferentiated melanoma which were negative for melanocytic markers, namely Melan-A/MART 1, HMB45, S100 protein, and SOX 10. ^12^^,^^13^ In these cases, molecular testing could help establish diagnosis detecting melanoma-compatible mutations like BRAF, NRAS and NF 1. ^13^ In the case of our patient, the diagnosis of melanoma was supported by the histopathological findings and typical immunohistochemical profile of both gastric and skin lesions, requiring no further investigations.

Metastatic melanoma undoubtedly carries a poor prognosis. Distant metastatic burden has been recognized as an important prognostic indicator. Plus, factors such as non-pulmonary visceral metastases and elevated serum lactate dehydrogenase (LDH) have been associated with an even more dismal survival, resulting in a one-year survival rate of only 33%. ^14^^,^^15^ In the above-mentioned systematic review of gastric melanoma metastases, the median survival time was 3 months, with 16% and 4% survival rates at 1 year and 2 years respectively. ^9^ There was also a significantly lower survival in case of multiple gastric lesions compared to single metastasis.

GI resections have been performed in localised metastasis, with evidence of possible prolonged remission, or as an emergency procedure for complicated cases like GI bleeding or perforation. ^8^^,^^16^ However, our patient presented extensive metastatic disease and hence was not a proper candidate for surgery. Patients with metastatic melanoma are more suited for systemic treatment, especially as in recent years, immunotherapy has shown promising results in improving survival, becoming the recommended first-line treatment. ^17^ Nevertheless, immunotherapy drugs were not available at the time, and our patient general status worsened rapidly preventing chemotherapy treatment.

In summary, this case report provides a comprehensive documentation of the clinical, endoscopic, histological, and immunohistochemical features of gastric metastasis from melanoma, which is a rare occurrence. The strength of this report lies in its detailed presentation of these aspects, contributing valuable insights into the diagnosis of such metastases. However, the limitations of this study include a short follow-up period and the absence of specific treatment due to the patient's overall condition and the unavailability of immunotherapy.

## Conclusion

Metastases to the gastrointestinal tract are considered rare, particularly in the stomach. However, melanoma is known to be the most common cancer that metastasises to this site. This report presents a case where malignant cutaneous melanoma was initially uncovered by a gastric metastasis, confirmed through endoscopic, histopathological, and immunohistochemical findings. Importantly, a protruding gastrointestinal lesion with dark pigmentation observed during endoscopy should raise suspicion of melanoma and prompt a thorough skin examination to identify any potentially overlooked lesions. Histopathological assessment and immunohistochemistry are the gold standards for diagnosis. For widespread disease, palliative systemic treatment, including chemotherapy or immunotherapy, is preferred, while surgery is typically reserved for selected oligometastatic cases. Despite these treatments, the prognosis remains poor.

## Consent

Written informed consent for publication of their clinical details and/or clinical images was obtained from the patient.

## Data Availability

No data are associated with this article. Zenodo: CARE checklist for ‘Case Report: Gastric Metastasis revealing a Disseminated Skin Melanoma: A Case Report and Literature Review’. DOI: https://zenodo.org/doi/10.5281/zenodo.13387861 ^18^ Data are available under the terms of the Creative Commons Zero “No rights reserved” data waiver (CC0 1.0 Public domain dedication).
