# Case Report: Overlooked Skin Melanoma Unveiled by Gastric Metastasis

**DOI:** 10.12688/f1000research.155815.4

**Published:** 2025-07-03

**Authors:** Ramzi Tababi, Amal Khsiba, Moufida Mahmoudi, Asma Ben Mohamed, Manel Yakoubi, Ghada Gharbi, Abir Chaabane, Emna Chelbi, Mouna Medhioub, Mohamed Lamine Hamzaoui

**Affiliations:** 1 Gastroenterology Department, Mohamed Taher Maamouri University Hospital Nabeul, Mrezga, Nabeul, Tunisia; 2 Pathology Department, Mohamed Taher Maamouri University Hospital Nabeul, Mrezga, Nabeul, Tunisia

**Keywords:** Melanoma, Gastric metastasis, Multi-organ metastases, Digestive endoscopy, Histopathology

## Abstract

**Background:**

Gastric metastasis from melanoma is rare and often presents as an unexpected finding, rarely revealing an underlying primary skin melanoma.

**Case presentation:**

We report a case of a 62-year-old male who presented with abdominal pain, dyspepsia, anorexia, and weight loss. On physical examination abdominal masses and hepatomegaly were detected. Radiological imaging showed widespread masses in the abdominal and thoracic regions. Upper gastrointestinal endoscopy identified an umbilicated protruded lesion with central dark pigmentation at the antro-fundic junction. Histopathological examination and immunohistochemical staining were consistent with melanoma. A subsequent rigorous skin examination uncovered a primary malignant skin melanoma. Due to worsening general condition, the patient received palliative hospice care.

**Conclusion:**

This report highlights the critical need for vigilant skin examination when dealing with widespread metastatic disease.

## Introduction

The incidence of melanoma is on the rise, particularly in developed countries with lighter skin populations, representing 1.7% of global cancer cases. ^1^ It also constitutes 10% of all skin cancers and remains the primary cause of death among these malignancies. ^2^ In fact, melanoma embodies a heterogeneous tumour group of distinct precursor cells, biological signature and presentations. ^1^^,^^2^ Nodular melanoma is notably a malignant subtype, known for its aggressive behaviour and accounting for 16% to 25.6% of invasive cutaneous melanoma. ^2^

The most frequent metastatic sites include the skin, lungs, liver, central nervous system and bones. Less frequently, metastases occur in the kidneys, adrenal glands, and gastrointestinal (GI) tract, with gastric involvement being particularly rare. ^3^

In this report, we present a rare case of gastric metastasis from melanoma that led to the discovery of the initially unnoticed primary skin lesion. We describe the endoscopic and histopathological findings, as well as the nonspecific clinical symptoms that prompted the diagnostic investigation.

## Case report

A 62-year-old Caucasian male patient, with a medical history of hypertension and coronary artery disease, presented with diffuse abdominal pain, along with dyspepsia, anorexia and weight loss for the past 6 months. Physical examination revealed abdominal tenderness and multiple fixed and hard nodules of the thoracic and abdominal walls, as well as in the Douglas pouch on rectal examination, suggestive of carcinomatosis. An enlarged, firm, and tender liver was also noted. No jaundice or palpable lymph nodes were observed.

Laboratory findings were within the normal range, notably for complete blood count and liver function tests.

**Figure 1.**
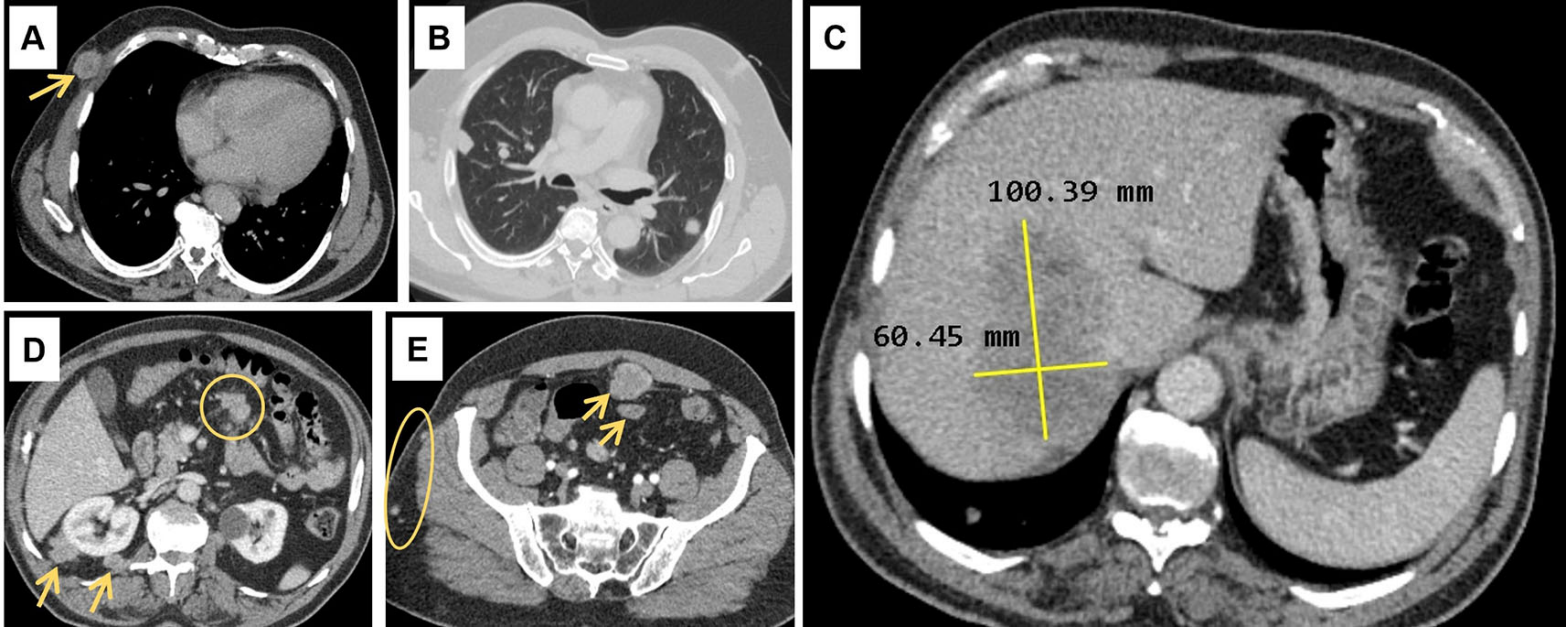
Computed tomography scan findings. (A): Subcutaneous soft tissue nodule of the anterior right thoracic wall (arrow). (B): Bilateral pulmonary nodules. (C): Hypodense hepatic mass in the right lobe on the portal phase, measuring 100 × 60 millimetres. (D): Intraperitoneal adenomegalies (circle) and heterogeneous nodules in the right perirenal retroperitoneal region. (E): Heterogeneous peritoneal nodules (arrows) and small subcutaneous soft tissue nodules in the right lateral abdominal wall (circle).

Abdominal ultrasound (US) showed a non-dysmorphic liver with a heterogenous lobulated hypoechoic mass, vascularised on Doppler, measuring 83 × 60 mm, associated with omental adipose tissue nodule of 37 mm, sharing the same characteristics. On the CT scan ( Figure 1), the hepatic mass was isodense, poorly defined, spanning segments V, VII and VIII, showing weak enhancement with contrast, measuring 100 × 60 mm. There were also multiple scattered tissue masses enhanced with contrast, located in the peritoneum, in the retroperitoneum, and sub-peritoneal, as well as in the left adrenal gland. The pancreas was normal. Plus, multiple solid pulmonary nodules were observed, involving all segments, alongside mediastinal and abdominal lymphadenopathies.

At this point, widespread metastases were the leading considered diagnosis based on the clinical context and radiological presentation. Alpha-fetoprotein and prostate specific antigen levels were normal.

**Figure 2.**
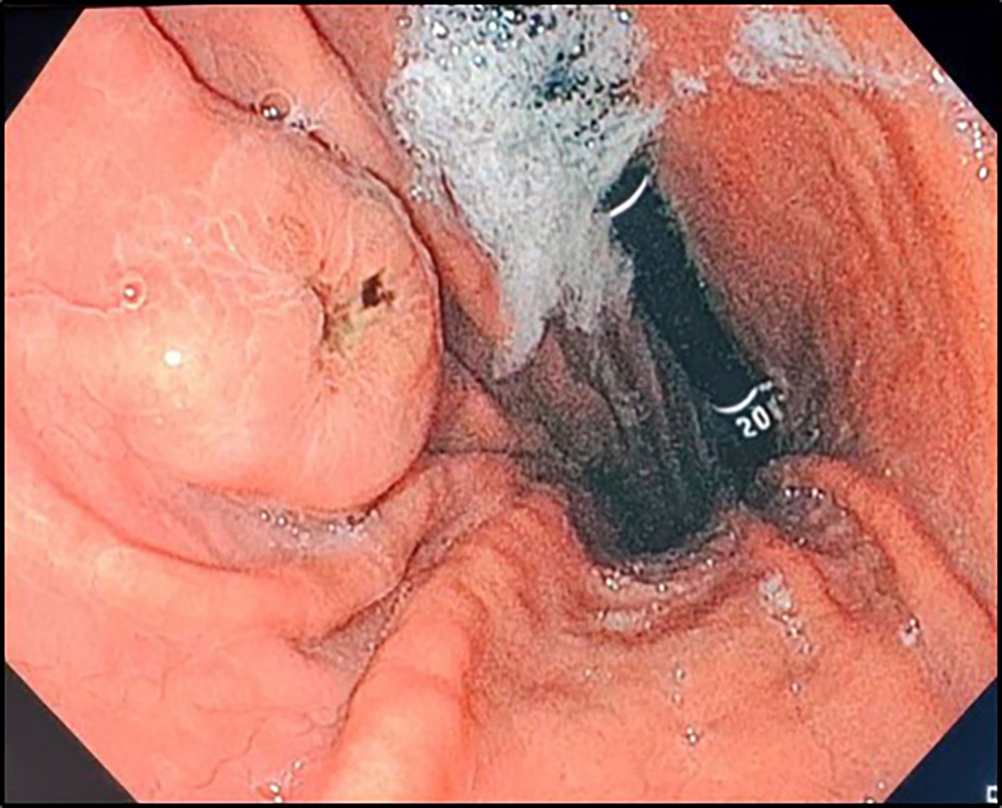
Upper gastrointestinal endoscopy findings. Retroflexion shows an elevated lesion at the antrum-fundus junction with a depressed centre, resembling a volcano appearance, with brown pigments.

**Figure 3.**
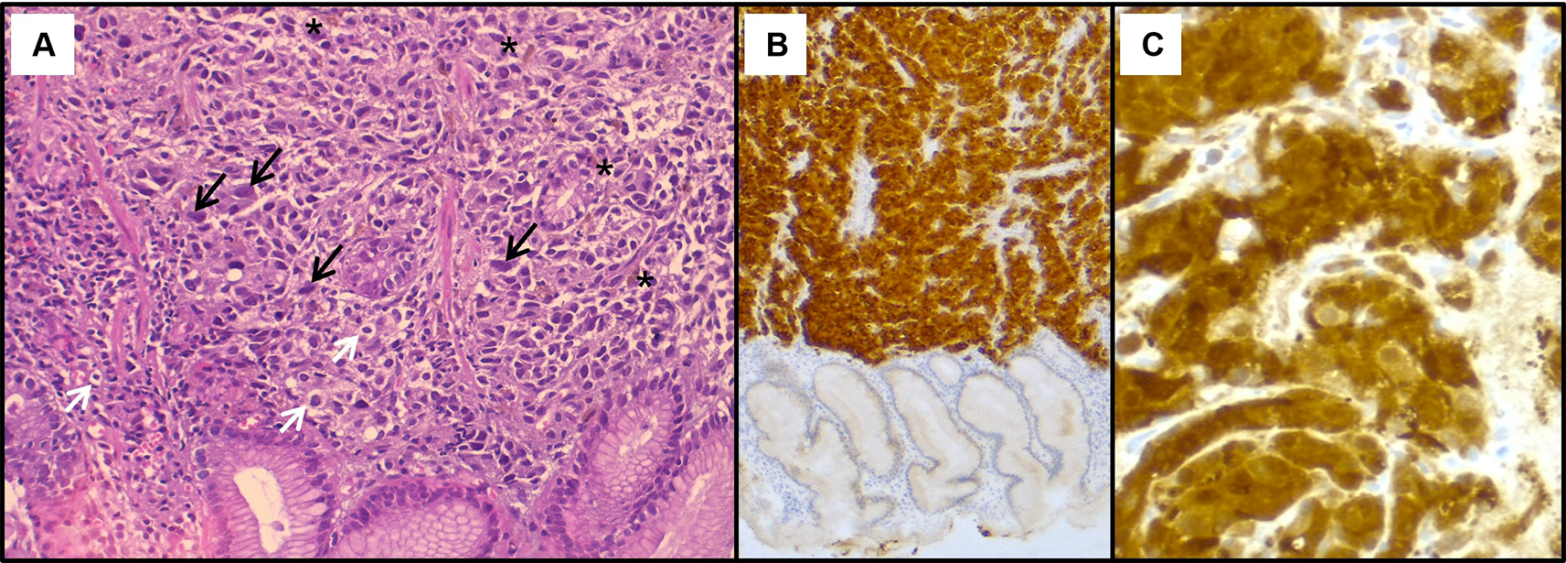
Histopathological findings of the gastric lesion biopsy. (A): Haematoxylin & eosin staining on x200 magnification shows malignant proliferation infiltrating the gastric mucosa. Tumour cells are pleomorphic and disaggregated, exhibiting marked nuclear atypia with hyperchromatic and sometimes monstruous nuclei (black arrows). Some larger cells with clear cytoplasm were present (white arrows). Deposits of brown pigments are also seen (*). Immunohistochemistry demonstrated positive staining with Melan-A (B) and PS 100 (C).

In the investigation for the primary cancer, and given the recent dyspepsia and epigastric pain, an upper gastrointestinal endoscopy was performed revealing a polypoid lesion at the antro-fundic junction measuring 20 mm in diameter, with a depressed and ulcerated centre, containing dark pigmentation ( Figure 2). The histological examination showed a tumour proliferation extensively invading the antro-fundic mucosa, composed of discohesive cells, with deposits of brownish pigments. Immunohistochemistry analysis revealed positive staining for melanocytic markers Melan-A, S100 protein and HMB45 ( Figure 3). Other markers were negative, namely pancytokeratin, TTF 1, CD 45, chromogranin, and synaptophysin. These findings were consistent with a gastric localisation a melanoma.

**Figure 4.**
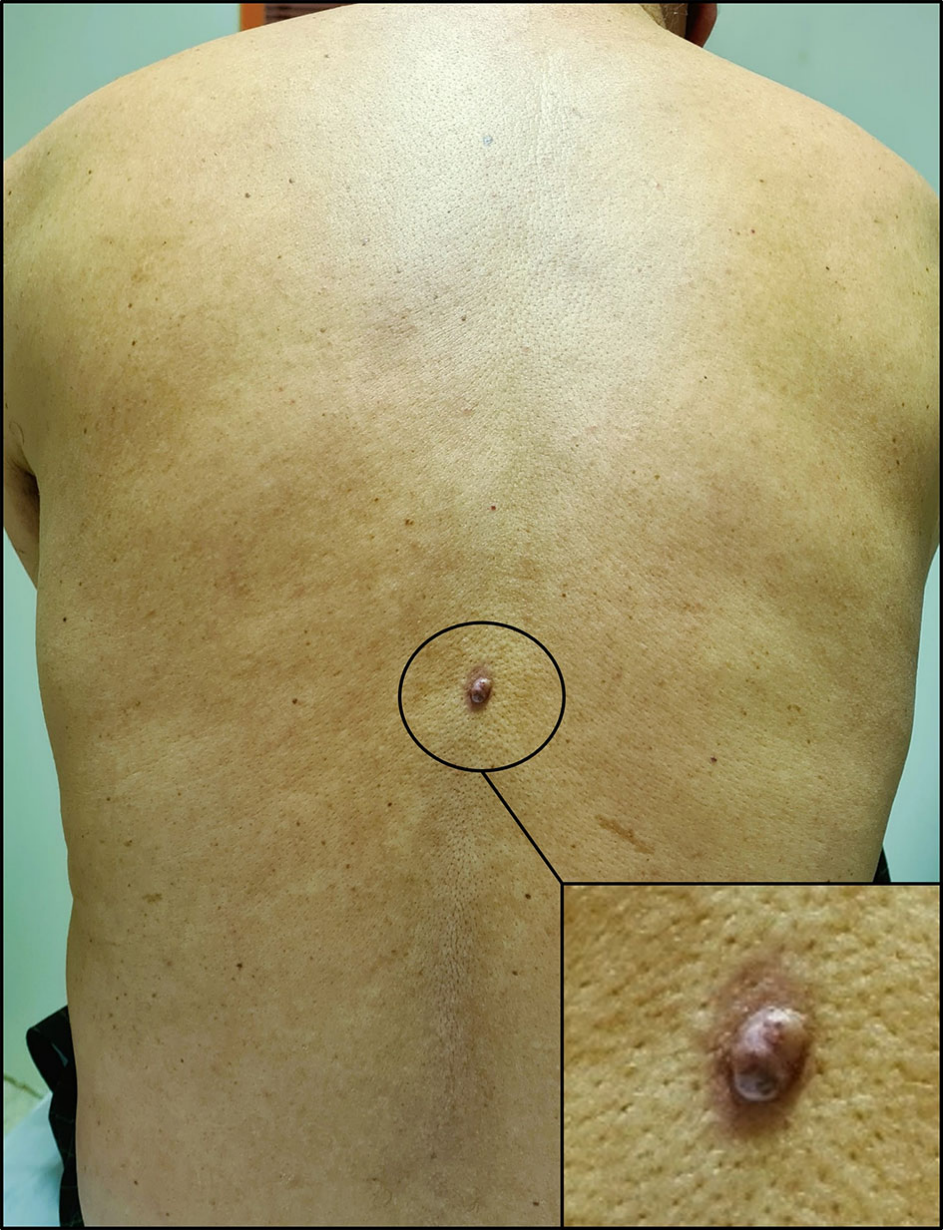
Cutaneous lesion in the back. It appears as a circumscribed dark pink nodule measuring 8 millimetres in diameter with areas of brown discoloration. The lesion also has a slightly irregular surface and a surrounding area of erythema.

With these results in mind, a thorough skin examination was thus undertaken unveiling a cutaneous lesion in the back, manifesting as a circumscribed pink nodule of 8 mm with brown discoloration ( Figure 4).

**Figure 5.**
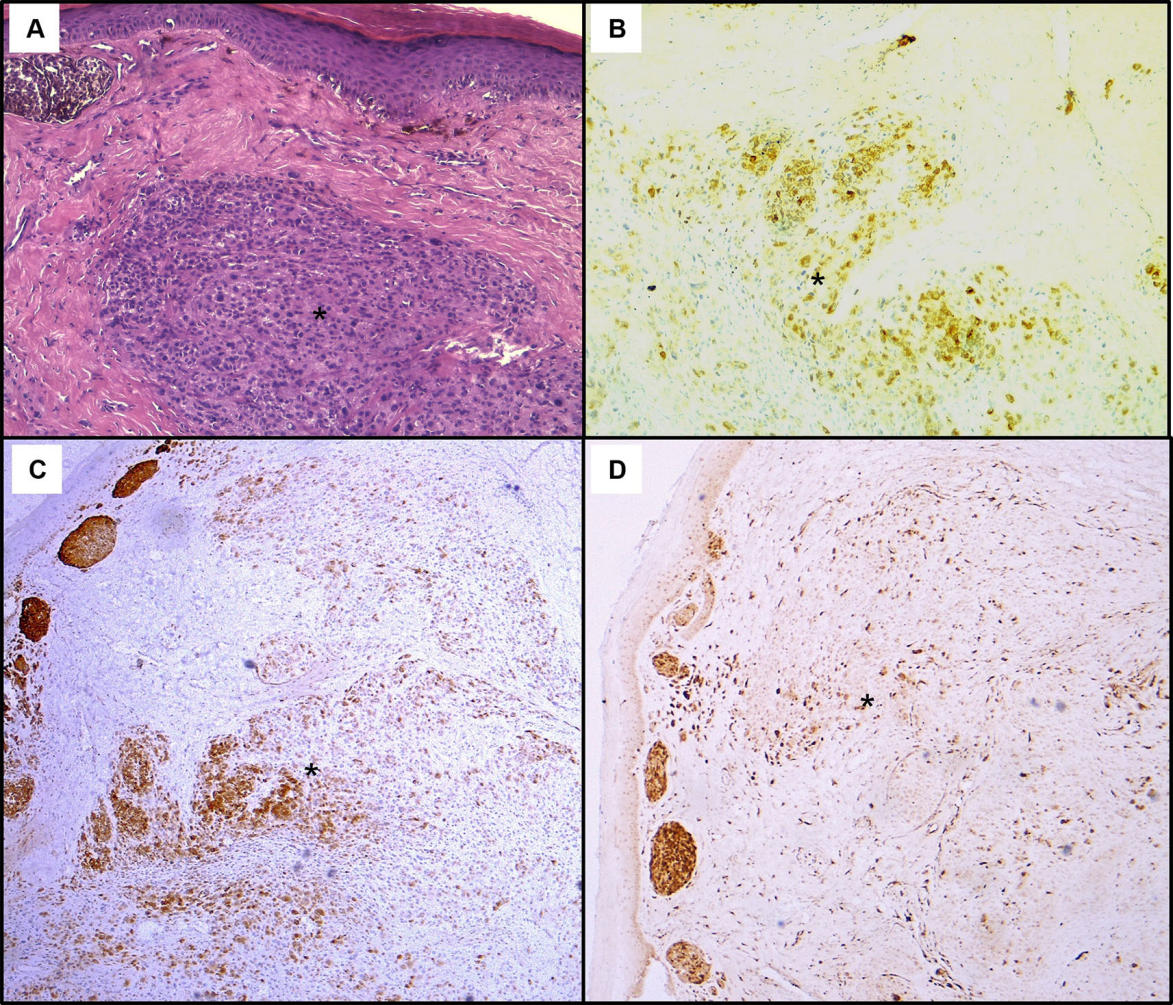
Histopathological findings of the skin lesion biopsy. (A): Haematoxylin & eosin staining on x100 magnification shows malignant cellular proliferation (*) in the dermis, composed of vaguely naevoid cells and larger cells with abundant eosinophilic cytoplasm, atypical pleomorphic nucleolated nuclei, and no junctional activity. On immunohistochemistry, tumour cells exhibit positive staining for HMB45 (B), Melan-A (C), and p16 (D).

Histological assessment of the skin lesion biopsy confirmed nodular melanoma, with immunohistochemistry showing positive staining for Melan-A, HMB45, and p16 ( Figure 5).

Ultimately, the diagnosis of malignant cutaneous melanoma with diffuse multi-organ metastases, particularly involving the stomach, was confirmed. Subsequently, the patient’s general condition deteriorated, precluding the initiation of planned palliative chemotherapy. Supportive care was provided, including pain management, with a documented follow-up of 6 months.

## Discussion

The stomach is an unusual metastatic site, accounting for 2.6% of secondary lesions, particularly from breast, lung, and melanoma. Melanoma frequently metastasizes to the GI tract, with cutaneous melanoma primarily affecting the upper GI tract, while uveal melanoma tends to spread to the liver. ^4^^,^^5^ An autopsy study of 216 patients with advanced malignant melanoma found the GI system to be the second most common metastatic site, after lymph nodes (73.6%) and lungs (71.3%). ^6^ In this series, GI metastases were often part of multiple-organ involvement (95%), with secondary gastric melanoma in 22.7% of cases. Liver metastases were observed in 58.3% of cases. Other GI metastases included the peritoneum (42.6%), pancreas (37.5%), small bowel (35.6%), spleen (30.6%), colon (28.2%), oral cavity, oesophagus (9.3%), and biliary tract (8.8%). A similar autopsy study of 100 patients with cutaneous melanoma found gastric involvement in 26%. ^7^ Despite these findings, GI metastases seem to be often subclinical, with clinical series showing much lower incidence, such as intestinal metastases in only 1% to 7% of cases. ^3^ Consistent with these reports, our patient presented with disseminated melanoma affecting multiple organs, including the lungs, liver, stomach, subcutaneous tissue, adrenal gland, peritoneum, and lymph nodes.

Also, gastric metastases from melanoma may be present at diagnosis or develop years later, indicating recurrence. ^8^

A recent systematic review by Reggiani et al. ^9^ included 113 patients with gastric melanoma metastases, predominantly from skin primary lesions (62%). Most patients were male (64%) with a median age of 63 years. Only 10% were asymptomatic, while common symptoms included bleeding (34.5%), abdominal pain (34.5%), anorexia and weight loss (23%), and nausea/vomiting (17.7%). Dyspepsia was noted in 5.3%. Gastric metastases were single in 42.5% and mostly located in the gastric body (60.2%). However, variability in lesion descriptions across studies prevented the identification of consistent endoscopic features.

Nelson et al. ^10^ proposed an endoscopic classification for GI melanoma metastases with three types: 1) melanotic nodules on normal folds with ulcerated tips; 2) elevated submucosal lesions with ulcerated centres, sometimes with visible melanin, often described as volcano-like or donut-shaped, ^9^ as seen in our case; and 3) mass lesions with necrosis and melanosis. Such protruding lesions, especially with brown deposits, should raise suspicion for gastric melanoma. Other descriptions include polypoid masses, small nodules, and black spots. ^5^^,^^8^^,^^11^

GI melanoma metastases can resemble primary gastric cancer or other gastric metastases, ^5^^,^^9^^,^^11^ making histopathological examination and immunohistochemistry essential for diagnosis. These metastases typically show positive staining for melanocytic markers, ^12^ as seen in both our gastric and skin lesions. However, phenotypic switching during tumour progression can cause loss of typical immunophenotypic features, such as Melan-A, HMB45, S100, and SOX 10. ^12^^,^^13^ In such cases, molecular testing for melanoma-related mutations like BRAF, NRAS, and NF1 may be necessary. ^13^ For our patient, the diagnosis was confirmed by histopathological findings and the typical immunohistochemical profile, without the need for further tests.

Metastatic melanoma has a poor prognosis, with distant metastatic burden being a key prognostic factor. Non-pulmonary visceral metastases and elevated serum lactate dehydrogenase are linked to worse survival, with a one-year survival rate of 33%. ^14^^,^^15^ In the above-mentioned systematic review of gastric melanoma metastases, the median survival was 3 months, with 16% and 4% survival rates at 1 and 2 years, respectively. ^9^ Survival was notably lower with multiple gastric lesions compared to a single metastasis.

GI resections have been performed in localised metastasis, with evidence of possible prolonged remission, or as an emergency procedure for complicated cases like GI bleeding or perforation. ^8^^,^^16^ However, our patient presented extensive metastatic disease and hence was not a proper candidate for surgery. Systemic treatments, particularly immunotherapy, are now the preferred first-line therapy for metastatic melanoma, showing promising survival benefits. ^17^ Unfortunately, immunotherapy was unavailable at the time, and the patient’s rapid decline precluded chemotherapy.

## Conclusion

GI metastases, particularly in the stomach, are rare, with melanoma being the most common cancer to spread to this site. This case highlights how a gastric metastasis revealed an underlying malignant cutaneous melanoma. A protruding gastric lesion with dark pigmentation during endoscopy should raise suspicion of melanoma, warranting a thorough skin examination for potentially overlooked lesions, which is also crucial in widespread metastatic disease. Histopathological and immunohistochemical analysis remain the gold standards for diagnosis. In cases of widespread metastases, palliative systemic treatment, including chemotherapy or immunotherapy, is preferred, with surgery reserved for selected oligometastatic cases. Despite treatment, the prognosis remains poor.

## Consent

Written informed consent for publication of their clinical details and/or clinical images was obtained from the patient.

## Data Availability

No data are associated with this article. Zenodo: CARE checklist for ‘Case Report: Gastric Metastasis revealing a Disseminated Skin Melanoma: A Case Report and Literature Review’. DOI: https://zenodo.org/doi/10.5281/zenodo.13387862 ^18^ Data are available under the terms of the Creative Commons Zero “No rights reserved” data waiver (CC0 1.0 Public domain dedication).
