# Supplementary material for: Case Report: Overlooked Skin Melanoma Unveiled by Gastric Metastasis
Source: F1000Res. 2025 Jul 3;13:1019. Originally published 2024 Sep 6. [Version 4] doi: 10.12688/f1000research.155815.4 (PMC12260486; doi:10.12688/f1000research.155815.4)
Supplement: Peer review history

# Reviewer report: Reviewer response for version 4

**DOI:** 10.5256/f1000research.184176.r396415

**Published:** 2025-07-14

**Authors:** Gerardo Ferrara

**Affiliations:** 1 G. Pascale Foundation National Cancer Institute IRCCS,, Naples, Italy

I have no further comments. The manuscript is now acceptable.

Are enough details provided of any physical examination and diagnostic tests, treatment given and outcomes?

Yes

Is the case presented with sufficient detail to be useful for other practitioners?

Yes

Is sufficient discussion included of the importance of the findings and their relevance to future understanding of disease processes, diagnosis or treatment?

Yes

Is the background of the case’s history and progression described in sufficient detail?

Yes

Reviewer Expertise:

NA

I confirm that I have read this submission and believe that I have an appropriate level of expertise to confirm that it is of an acceptable scientific standard.

# Reviewer report: Reviewer response for version 3

**DOI:** 10.5256/f1000research.176558.r357035

**Published:** 2025-01-08

**Authors:** Gerardo Ferrara

**Affiliations:** 1 G. Pascale Foundation National Cancer Institute IRCCS,, Naples, Italy

I have two queries:

 1. The authors should rewrite the following sentence :

 ". These metastases typically show melanocytic features, including cytological atypia, pleomorphism, and positive staining for melanocytic markers, ^12^ as seen in both our gastric and skin lesions. However, phenotypic switching during tumour progression can cause loss of typical immunophenotypic features, such as Melan-A, HMB45, S100, and SOX 10. ^12^^,^^13^ In such cases, molecular testing for melanoma-related mutations like BRAF, NRAS, and NF1 may be necessary. ^13^ "

 Cytologic atypia and pleomorphism are clues to malignancy and not clues to melanoma. Having said this, I agree that metastatic melanoma may 'dedifferentiate' (the Authors might add a reference) and lose some or even ALL the pan-melanocytic immunohistochemical markers, thereby requiring molecular investigation.

 2. The Authors should explain the meaning of the sentence 'who can see the elephant in the room?': something which is self-evident (in this case, the cutaneous primary) and is nevertheless at risk of being missed.

Are enough details provided of any physical examination and diagnostic tests, treatment given and outcomes?

Yes

Is the case presented with sufficient detail to be useful for other practitioners?

Yes

Is sufficient discussion included of the importance of the findings and their relevance to future understanding of disease processes, diagnosis or treatment?

Yes

Is the background of the case’s history and progression described in sufficient detail?

Yes

Reviewer Expertise:

Anatomic Pathology, Dermatopathology

I confirm that I have read this submission and believe that I have an appropriate level of expertise to confirm that it is of an acceptable scientific standard, however I have significant reservations, as outlined above.

# Reviewer report: Reviewer response for version 2

**DOI:** 10.5256/f1000research.175503.r349179

**Published:** 2024-12-30

**Authors:** Gerardo Ferrara

**Affiliations:** 1 G. Pascale Foundation National Cancer Institute IRCCS,, Naples, Italy

I have minor suggestions.

 1. The Authors should explain the title. Who can see the elephant in the room? this means that something self-evident is at risk of being missed.

 2. This case highlights the need for a rigorous skin examination when dealing with widespread metastatic disease

 3. "Disintegrated cells" is funny. Discohesive is better

 4. Is Figure 3B a MART1 stain. Looks like an S100

 5. I think that liver mets must be not included among the mets to the GI apparatus

 6. Discussion, para 2 after ref 8: please delete

 7. Discussion, Para 8: please delete.

Are enough details provided of any physical examination and diagnostic tests, treatment given and outcomes?

Yes

Is the case presented with sufficient detail to be useful for other practitioners?

Yes

Is sufficient discussion included of the importance of the findings and their relevance to future understanding of disease processes, diagnosis or treatment?

Yes

Is the background of the case’s history and progression described in sufficient detail?

Yes

Reviewer Expertise:

Anatomic Pathology, Dermatopathology

I confirm that I have read this submission and believe that I have an appropriate level of expertise to confirm that it is of an acceptable scientific standard, however I have significant reservations, as outlined above.

# Response

**Published:** 2025-01-02

**Authors:** Ramzi Tababi

**Affiliations:** Gastroenterology, Mohamed Taher Maamouri University Hospital Nabeul, Mrezga, Nabeul, Tunisia

We appreciate the valuable suggestions and have addressed the comments as follows:

 1) The title has been revised to "Can You See the Elephant in the Room?" to make the metaphor clearer and more understandable.

 2) We emphasised the importance of a rigorous skin examination in widespread metastatic disease in both the abstract and conclusion.

 3) We have changed "disintegrated cells" to "discohesive cells".

 4) We have double-checked and can confirm that Figure 3B shows a MART1 stain.

 5) Although the liver is generally considered part of the digestive system and is often grouped with GI organs in the literature (including the reference we cited), we have, as suggested, placed it in a separate sentence to avoid any confusion.

 6+7) We have deleted these paragraphs (no.2 and 8) as recommended.

# Reviewer report: Reviewer response for version 1

**DOI:** 10.5256/f1000research.171029.r322061

**Published:** 2024-11-06

**Authors:** Hanen Jaziri

**Affiliations:** 1 University of Sousse, Sousse, Tunisia

- This an original case report because gastric metastasis of melanoma is very rare and uncommon  and in this case the metastases revelled the skin melanoma 
- Vigilant skin examination is very important because the prognosis of this tumour is worse 
- This case is illustrated by computed tomography scan endoscopic and histological fundings
- No modification is needed

Are enough details provided of any physical examination and diagnostic tests, treatment given and outcomes?

Yes

Is the case presented with sufficient detail to be useful for other practitioners?

Yes

Is sufficient discussion included of the importance of the findings and their relevance to future understanding of disease processes, diagnosis or treatment?

Yes

Is the background of the case’s history and progression described in sufficient detail?

Yes

Reviewer Expertise:

gasroenterology

I confirm that I have read this submission and believe that I have an appropriate level of expertise to confirm that it is of an acceptable scientific standard.

# Response

**Published:** 2024-12-01

**Authors:** Ramzi Tababi

**Affiliations:** Gastroenterology, Mohamed Taher Maamouri University Hospital Nabeul, Mrezga, Nabeul, Tunisia

Thank you for your positive feedback. We appreciate your recognition of the rarity and significance of the case, as well as the importance of vigilant skin examination in such patients. We are also glad that the use of imaging and histological findings was well-illustrated.

# Reviewer report: Reviewer response for version 1

**DOI:** 10.5256/f1000research.171029.r325913

**Published:** 2024-11-05

**Authors:** Gerardo Ferrara

**Affiliations:** 1 G. Pascale Foundation National Cancer Institute IRCCS,, Naples, Italy

Overall the manuscript seems to be worth of indexing, because physical examination of the skin may be neglected when a patient discloses severe systemic symptoms. I have the following suggestions:

 1. I suggest the Authors to rethink of the title by looking for some appealing features of the case (e.g.: can you see the elephant in the room?)

 2. The manuscript must be shortened; no need to define melanoma; no need to itemize the cytomorphological feature of the cells on gastric biopsy

 3. Besides being too long, the discussion is untidy. I suggest grouping clinical studies on one side, and autopsy studies on the other one

Are enough details provided of any physical examination and diagnostic tests, treatment given and outcomes?

Yes

Is the case presented with sufficient detail to be useful for other practitioners?

Yes

Is sufficient discussion included of the importance of the findings and their relevance to future understanding of disease processes, diagnosis or treatment?

Yes

Is the background of the case’s history and progression described in sufficient detail?

Yes

Reviewer Expertise:

Anatomic Pathology, Dermatopathology

I confirm that I have read this submission and believe that I have an appropriate level of expertise to confirm that it is of an acceptable scientific standard, however I have significant reservations, as outlined above.

# Response

**Published:** 2024-12-01

**Authors:** Ramzi Tababi

**Affiliations:** Gastroenterology, Mohamed Taher Maamouri University Hospital Nabeul, Mrezga, Nabeul, Tunisia

We greatly appreciate your constructive feedback on our manuscript. We have carefully considered your suggestions and made the following revisions:

1. **Title Revision**: In response to your suggestion, we have revised the title to make it more engaging and reflective of the core aspect of the case. We believe the new title highlights the critical role of the gastric metastasis in revealing the previously undetected cutaneous melanoma, aligning with the key feature of the case.
2. **Manuscript Shortening**: As recommended, we have shortened the manuscript by removing the definition of melanoma and the detailed itemization of the cytomorphological features in the gastric biopsy section. Several sentences have also been reformulated in order to reduce the word count. These changes have streamlined the manuscript, keeping the focus on the essential points of the case.
3. **Discussion Structure**: We have reorganized the discussion section as per your suggestion. We have followed a structured approach in presenting this section, starting with the frequency of GI and gastric metastasis (referencing two autopsy studies in the first paragraph to highlight the prevalence of GI involvement). Subsequent sections discuss (in order) clinical presentation, endoscopic findings, histopathological findings, prognosis, treatment, and finally, the strengths and limitations of the case. Autopsy studies were only included in the first paragraph to support the epidemiology of GI metastasis, while the remainder of the references are clinical studies. To improve conciseness and readability, we have streamlined the discussion by removing redundant sentences without compromising key points.

We believe these revisions have enhanced the clarity and focus of the manuscript. Thank you once again for your valuable suggestions, which have significantly improved our work.
